# Distributed cortico-subcortical networks enable robust speech state detection from sparse intracranial recordings

**DOI:** 10.3389/fnins.2026.1816455

**Published:** 2026-05-08

**Authors:** Chen Feng, En Zhang, Yifei Jia, Zhoule Zhu, Junming Zhu, Di Wu, Kedi Xu

**Affiliations:** 1 College of Biomedical Engineering and Instrument Science, Zhejiang University, Hangzhou, China; 2 Department of Neurosurgery, The Second Affiliated Hospital Zhejiang University School of Medicine, Hangzhou, China; 3 State Key Laboratory of Cognitive Neuroscience and Learning and IDG/McGovern Institute for Brain Research, Beijing Normal University, Beijing, China; 4 School of Engineering, Westlake University, Hangzhou, China; 5 School of Intelligence Science and Engineering, College of Artificial Intelligence, Harbin Institute of Technology, Shenzhen, China

**Keywords:** electrocorticography, intracranial recordings, natural speech, neural decoding, neurolinguistics, SEEG, speech state

## Abstract

**Introduction:**

Accurate and reliable detection of speech state transitions is a prerequisite for practical speech brain–computer interfaces (BCIs). While cortical language areas have been extensively studied, it remains unclear whether speech onset information is exclusively localized to these regions or distributed across a broader cortico-subcortical network. Here, we investigated the feasibility of decoding speech state transitions using sparse stereo-electroencephalography (SEEG) recordings that sample both cortical and subcortical structures.

**Methods:**

Four Mandarin-speaking epilepsy patients undergoing clinical SEEG monitoring performed a sentence-reading task. Neural signals were segmented and labeled as rest or speech based on acoustic onset. A convolutional neural network was trained to classify speech states using broadband or high-gamma features derived from different anatomical channel subsets. We further evaluated continuous decoding performance, model robustness to channel dropout, and the specific contributions of different brain regions.

**Results:**

Speech state decoding accuracy exceeded chance level (50%) in all participants, with peak single-participant accuracies surpassing 90%. Models integrating both cortical and subcortical signals generally outperformed those restricted to a single anatomical domain. Notably, broadband signals yielded higher classification accuracy than high-gamma features. In continuous decoding simulations, performance remained above chance, although reduced relative to discretized evaluation. Crucially, decoding accuracy was robust to random channel reduction (up to 50%) and remained above 70% even after excluding classical speech-related cortical regions. Contribution analyses indicated participant-specific patterns of model sensitivity, with relatively higher contributions observed in frontal regions and the thalamus in multiple participants.

**Discussion:**

These findings support the hypothesis that speech state information is represented in a distributed cortico-subcortical network rather than being confined to canonical language areas. The robustness of decoding performance despite channel reduction and regional exclusion suggests that sparsely sampled SEEG data can effectively drive speech detection modules. This study demonstrates the feasibility of utilizing deep brain recordings for speech BCIs, offering a pathway toward more stable and generalized implantable systems. Moreover, such autonomous speech state detection may also serve as an ethical safeguard, ensuring that neural language decoding is activated only during intended communicative acts.

## 1 Introduction

Human speech production is a complex physiological process emerging from coordinated activity across distributed cortical and subcortical networks. Beyond the classical modular view of Broca’s and Wernicke’s areas (Broca, 1861; Geschwind, 1970), modern neuroimaging and electrophysiology have mapped a vast, multi-level hierarchy spanning the front-temporal cortex and critical subcortical nodes (Hickok and Poeppel, 2007; Price, 2012). This network integrates high-level linguistic planning with the fine-grained motor control of the vocal tract (Duffau et al., 2014). This distributed neural architecture provides the fundamental basis for restoring communication via brain–computer interfaces (BCIs).

Recent breakthroughs in language neuroprostheses have transitioned from decoding isolated phonemes to synthesizing continuous, high-fidelity speech (Anumanchipalli et al., 2019; Moses et al., 2021; Willett et al., 2023; Metzger et al., 2023; Leonard et al., 2024; Silva et al., 2024a; Card et al., 2024; Silva et al., 2024b; Kunz et al., 2025; Feng et al., 2025; Wairagkar et al., 2025; Littlejohn et al., 2025). However, a persistent yet under-addressed challenge for real-time clinical deployment is the “speech state detection” problem, the ability to autonomously distinguish intended speech from neural silence or background cognitive activity. Current high-performance BCIs often necessitate deliberate, user-initiated triggers, requiring patients to manually signal the start and end of each utterance. This dependency not only imposes a significant cognitive overhead but also disrupts the fluid, spontaneous nature of human conversation (Anumanchipalli et al., 2019; Moses et al., 2021; Willett et al., 2023; Metzger et al., 2023; Feng et al., 2025; Wairagkar et al., 2025; Littlejohn et al., 2025). Without a robust, autonomous gating mechanism, BCIs are prone to “false activations” during non-speech activity and high latency during onset, fundamentally decoupling the neural intention from the machine’s execution. To achieve a truly seamless neuroprosthesis, the system must transparently decode the transition between rest and active production without requiring auxiliary user control.

While high-density electrocorticography (ECoG) has set the gold standard for speech decoding via cortical high-gamma activity (HGA), its window into the brain is inherently restricted to focal hubs on the lateral cortical surface (Leonard et al., 2024; Silva et al., 2024a; Moses et al., 2021; Anumanchipalli et al., 2019). This localized focus leaves a vast portion of the speech-related neural architecture, particularly deep-seated folds and subcortical nuclei, entirely untapped for real-time BCI applications. It remains an open question whether robust speech state detection must rely exclusively on these high-intensity surface signals, or if the transition between rest and speech is represented more broadly across a multi-level hierarchy. Transitioning from a hub-centric approach to one that explores the full anatomical landscape of the speech network could fundamentally redefine how we detect neural intention.

Stereo-electroencephalography (SEEG) provides the ideal platform to move beyond the cortical surfaces (Bancaud et al., 1970; Bartolomei et al., 2018; Bartolomei et al., 2017). By utilizing electrodes implanted perpendicular to the brain surface, SEEG enables simultaneous sampling of both cortical and deep subcortical structures (Li et al., 2025; Feng et al., 2025; Verwoert et al., 2022; Wu et al., 2024; Angrick et al., 2021; Verwoert et al., 2025). This study investigates whether such a sparse, yet brain-wide distributed sampling can support robust state detection that is less dependent on any single cortical area. We hypothesize that the neural signatures of speech onset are not localized events but are distributed across a coordinated cortical–subcortical ensemble. Tapping into this distributed network may enable an “always-on” gating mechanism that operates autonomously, alleviating the need for user-mediated triggers and moving closer to a seamless, natural language prosthesis.

In this work, we demonstrate that human speech states can be decoded with high fidelity from sparse, whole-brain stereo-electroencephalography (SEEG) signals, achieving a peak decoding accuracy of over 90%. Our results reveal that the neural information necessary for speech state detection is not confined to traditional cortical hubs but is instead robustly distributed across a multi-level hierarchy of cortical and subcortical structures. The decoding framework maintained robust performance exceeding 70% accuracy, even when classical eloquent areas were entirely excluded.

## 2 Materials and methods

### 2.1 Participants

Following approval from the Ethics Committee of the Second Affiliated Hospital of Zhejiang University School of Medicine (2024-0174), four epilepsy patients (2 males and 2 females; ages 20, 25, 19, and 19 for P1–P4) were recruited for this research. Informed consent was obtained from all participants prior to their involvement. These native Mandarin speakers, all left-hemisphere dominant, underwent stereoelectroencephalography (SEEG) electrode implantation (12, 8, 11, and 13 electrodes, respectively) for a 7–14 day monitoring period as part of their routine clinical care. The electrode implantation configurations, including site selection and lead count, were determined exclusively by clinical requirements for epilepsy monitoring.

**Figure 1:**
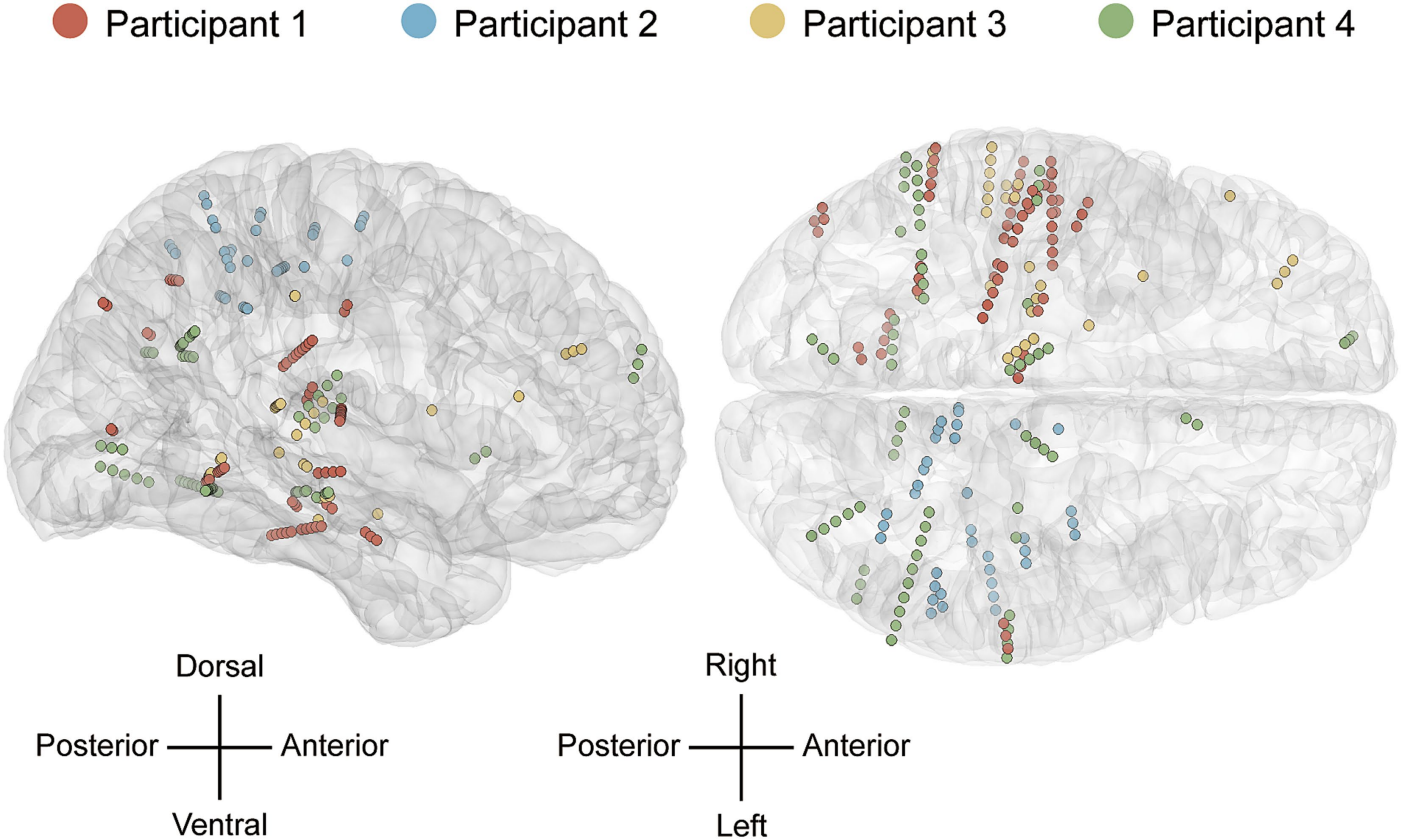
Anatomical localization of SEEG channels. Distribution of electrode contacts across the four participants, projected onto a transparent MNI template. Distinct colors denote contacts from different participants.

To determine electrode placement, preoperative T1-weighted MRI scans were coregistered with postoperative CT images using SPM12. Cortical pial surfaces were reconstructed using the FreeSurfer neuroimaging suite to anatomically localize each electrode contact (Fischl, 2012). Only contacts localized within cortical or subcortical gray matter were included in subsequent analyses, while contacts identified within white matter or cerebrospinal fluid were excluded to avoid contamination from volume conduction and non-neuronal signals. For population-level visualization, electrode coordinates were normalized to the Montreal Neurological Institute (MNI) template (Mazziotta et al., 2001), as illustrated in Figure 1.

### 2.2 Study design

**Figure 2:**
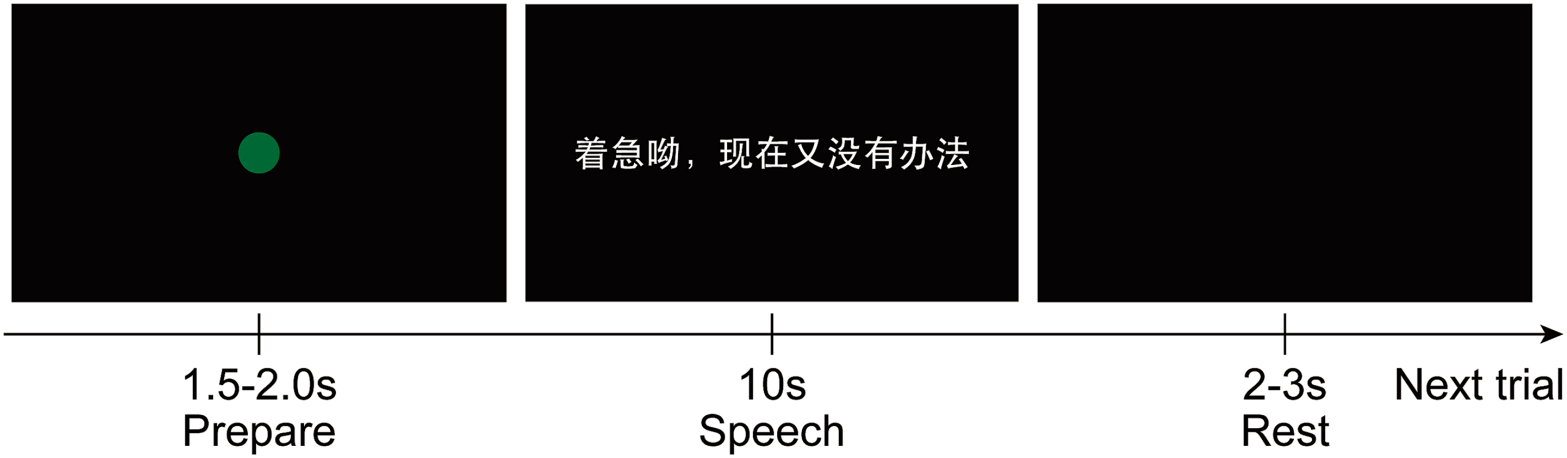
Schematic illustration of the experimental paradigm. Each trial consists of three sequential phases: (1) a preparation phase (1.5–2.0 s) indicated by a green fixation circle; (2) a speech production phase (10 s) during which a Mandarin sentence (e.g., “着急呦，现在又没有办法”) is displayed for the participant to read aloud; and (3) a rest phase (2.0–3.0 s) with a blank screen before the onset of the next trial.

Participants were instructed to read aloud 100 Chinese sentences, with each sentence repeated three times. Sentences were displayed in black text on a white background, presented on a 32-inch monitor positioned approximately 60 cm from the participant. As illustrated in Figure 2, each trial consisted of three distinct phases: (i) a preparation period lasting 1.5–2.0 s; (ii) a reading phase, during which a sentence was read aloud in Mandarin Chinese within 10 s; and (iii) a rest period of 2–3 s. All evaluation data were collected within a single experimental session on the same day.

### 2.3 Data acquisition and preprocess

#### 2.3.1 Neural signal

Neural activity was recorded at a sampling frequency of 2,000 Hz using a professional multi-channel electrophysiological system (Neurofax EEG-1200, Nihon Kohden, Japan). Speech-related channel selection was performed by comparing the PSD of speech versus silence intervals using Welch’s method. Specifically, we conducted paired *t*-tests on the mean power (4–150 Hz) for each non-white matter channel, contrasting the signal after acoustic onset with the preceding silence. Significance was defined at *p* < 0.05 after FDR correction via the Benjamini-Hochberg method. To ensure decoding purity, channels situated in visual processing areas like the occipital lobe were discarded regardless of their significance. Figure 1 illustrates the anatomical locations of the resulting channel sets, comprising 63, 36, 30, and 66 channels for the respective participants, with distinct colors representing each individual.

**Figure 3:**
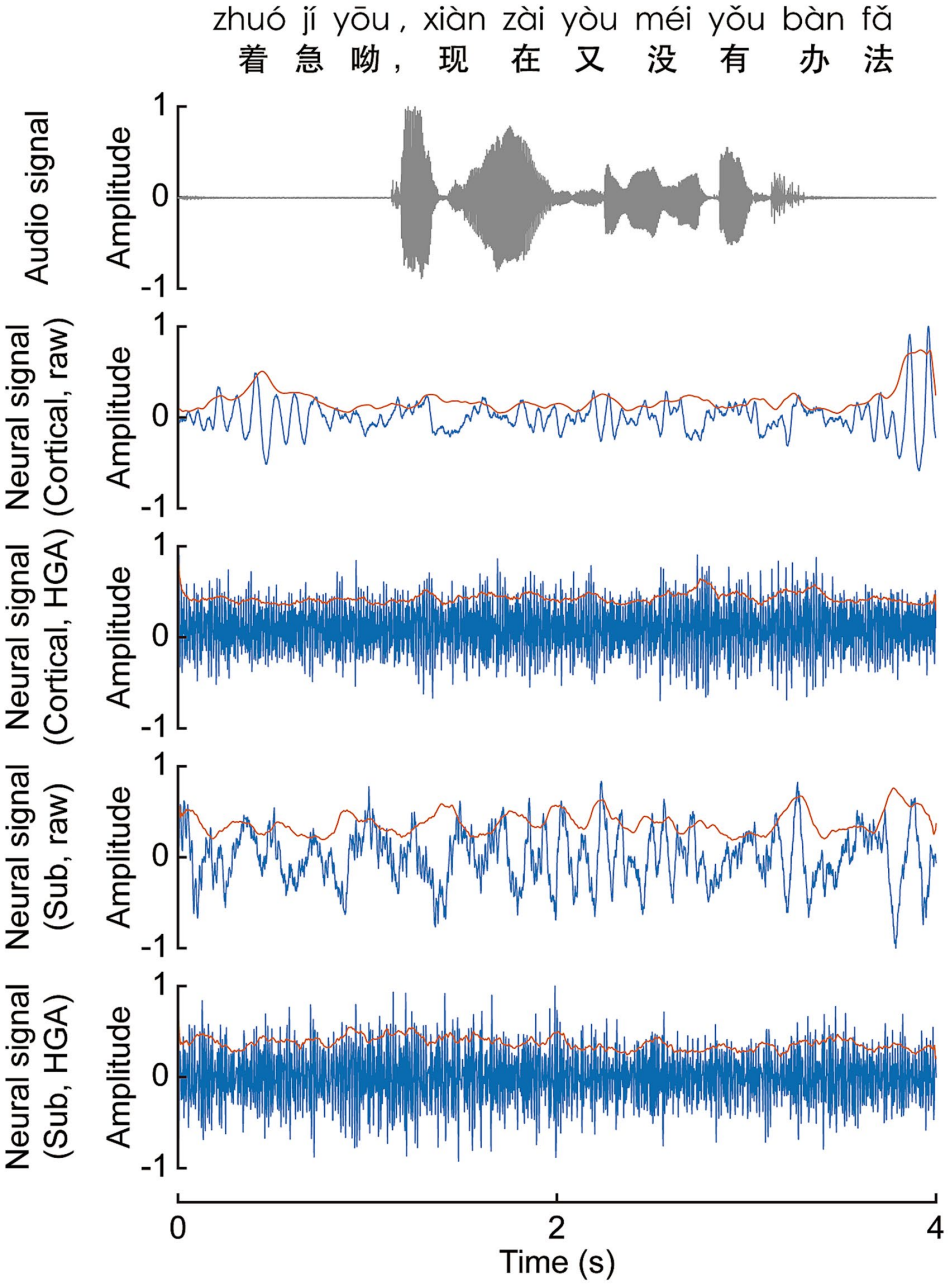
Representative recordings of synchronized acoustic and neural signals during Mandarin speech. The top panel displays the acoustic waveform for the spoken Mandarin sentence: “着急呦，现在又没有办法” (Zháojí yōu, xiànzài yòu méiyǒu bànfǎ). Subsequent panels show time-aligned neural activity from representative cortical and subcortical channels. For each region, both the voltage fluctuations (blue) and the extracted envelopes (orange) of the raw signal and HGA are presented over a 4-s window. All signals are normalized to an amplitude range of [−1, 1].

To benchmark the speech onset detection model against different signal characteristics, the raw neural signals were processed using two distinct filtering strategies. First, to capture standard broadband activity, signals were subjected to a normal anti-aliasing low-pass filter (200 Hz, raw in Figure 3). Second, to evaluate the model’s performance on rapid neural fluctuations, a specific band-pass filter (75–150 Hz) was applied to obtain HGA. Both signal streams subsequently underwent a series of notch filters (50, 100, 150, and 200 Hz) to eliminate powerline interference (Figure 3). Channels characterized by a low signal-to-noise ratio or abnormal physiological artifacts were identified during visual inspection and excluded from further analysis. Finally, the signals underwent a standardization process using z-score standardization and the continuous data were segmented into trials and aligned with the onset of individual Chinese character pronunciations based on task annotations. Following preprocessing, signals were down sampled from 2,000 Hz to 500 Hz to reduce computational load while preserving the frequency content of interest (up to 200 Hz).

#### 2.3.2 Audio signal

A directional microphone (Audio-Technica) was used to record audio at 44.1 kHz. High accuracy synchronization between audio and SEEG recordings was achieved via a hardware trigger signal (TTL pulse) generated by the stimulus presentation software at the onset of each trial (Tang et al., 2025). To mitigate ambient noise within the clinical ward, temporary soundproof barriers were deployed around the patient. Figure 3 illustrates the acoustic waveform of the spoken Mandarin sentence: “着急呦，现在又没有办法.” To safeguard neural recordings against audio interference, we implemented two decoupled hardware setups for separate data collection. Before subsequent analysis, the independence of these signals was validated by calculating their correlation across synchronized, randomly selected 10-min epochs. These tests (see Supplementary Figure S1) demonstrated no evidence of signal contamination, confirming the efficacy of our isolated recording approach (Roussel et al., 2020).

The audio data underwent a rigorous quality control process: recordings containing significant noise or pronunciation errors were manually identified and excluded, along with their corresponding neural signals. For model training and evaluation, task annotations including precise phonetic labeling were required, specifically marking the onset and offset of each character. This was achieved through a hybrid approach: initial automated segmentation via PRAAT software (Boersma, 2011), followed by manual verification and refinement by three phonetics experts to ensure annotation accuracy. Finally, to facilitate binary state classification, the labeled audio and aligned neural signals were categorized into two distinct phases.

The “rest state” was defined as any signal duration prior to the onset of the first syllable component. Conversely, the “speech state” was defined as the interval inclusive of and following the first syllable component, encompassing all subsequent vocalizations and intra-sentence pauses. This binary labeling scheme was motivated by the intended application as a front-end gating module for speech BCIs. In naturalistic connected speech, intra-sentence pauses serve essential functions including respiratory coordination, prosodic structuring, and lexical planning, and the speaker remains in an active communicative state throughout the utterance. From a practical BCI standpoint, a speech state detector should remain activated throughout the entire utterance—including brief pauses—to avoid premature system disengagement that would disrupt fluent communication. This labeling strategy prioritizes ecological validity and clinical utility over signal homogeneity, aligning the classification task with the real-world operating conditions of a speech BCI.

### 2.4 Speech states detection

Neural signal from the selected C input channels (where C denotes the channel count) corresponding to each sentence was sliced into non-overlapping segments of 100 ms. Given the 500 Hz sampling rate established during preprocessing, each temporal segment corresponds to *T* = 50 discrete sample points, resulting in an input tensor of dimension (C, T) for each segment. With state labeling from audio signals, the neural signal segments could be labeled as “rest state” and “speech state” segments. We adopt a simple yet effective plain convolutional neural network (CNN) architecture as the backbone for speech state decoding. The detailed architecture of the network is provided in Table 1. The participants’ pre-processed SEEG of input channels C is first passed to a stem layer containing a 1-D convolution layer. The 1-D convolution layer adopts a kernel size of 15 × 1, a stride of 4, and an output channel number of 32. Each convolution block consists of a 1-D convolution layer and a max pooling layer. The number of the output channel of the three blocks is set to 64, 128, 256, respectively. Then, we adopt a global average pooling layer and a fully connected layer to map the output of the last block to fit the binary prediction target. The objective function to train the network is defined to be binary cross-entropy loss between predicted state and ground-truth state.

**Table 1:** Detailed architecture specifications for the convolutional neural network utilized in this work.

Module	ConvNet
Stem	15×1,32,stride4
Block 1	$[\begin{array}{c} 7\times 1,64\\ \text{MaxPool},\text{stride}\;2 \end{array}]\times 1$
Block 2	$[\begin{array}{c} 5\times 1,128\\ \text{MaxPool},\text{stride}\;2 \end{array}]\times 1$
Block 3	$[\begin{array}{c} 5\times 1,256\\ \text{MaxPool},\text{stride}\;2 \end{array}]\times 1$

### 2.5 Experimental setup

The experiments were conducted using PyTorch on a NVIDIA A800 GPU. The dataset consisted of 300 sentence trials (100 unique sentences × 3 repetitions). To prevent information leakage, data were split at the sentence level rather than at the trial level. Specifically, all repetitions of a given sentence were assigned exclusively to either the training set or the evaluation set, ensuring that no sentence content appeared in both sets. The split ratio was 80% training and 20% evaluation, and this procedure was repeated using 20 distinct random seeds. We carry out experiments on all four participants with the same experimental setup and was repeated on both standard and high gamma activity neural signals. The models were trained using the Adam optimizer, with a base learning rate of lr = 0.001. Momentum parameters are set to $\beta_{1}=0.9$ , $\beta_{2}=0.999$ , along with weight decay of 0.005 and a cosine decay approach was adopted for the learning rate schedule. To mitigate overfitting, a dropout layer with a rate of 0.1 is applied after the max pooling layer in each convolutional block. The training was conducted over 80 epochs, incorporating an early stopping criterion based on a five-epoch non-improvement threshold on the validation set. The complete list of parameters used for model training is available in Table 2.

**Table 2:** Optimizer setup for speech state prediction.

Configuration	ConvNet
Training epochs	80
Optimizer	Adam
Base learning rate	0.001
Weight decay	0.005
Optimizer momentum	*β*_1_ = 0.9, *β*_2_ = 0.999
Batch size	48
Learning rate schedule	cosine decay
Dropout ratio	0.1

### 2.6 Continuous decoding

To evaluate the model’s capability for continuous decoding in a quasi-real-time setting, a separate validation protocol was established. A subset of 26 sentences was selected from the evaluation dataset based on the following criteria: (i) adequate sentence length to provide sufficient rest-to-speech transitions, (ii) clean pronunciation quality as verified during the audio quality control process, and (iii) absence of significant noise artifacts in the corresponding neural recordings. Critically, all selected sentences and their three repetitions belonged exclusively to the held-out evaluation set, with no overlap in sentence content with the training data, consistent with the sentence-level splitting protocol described in Section 2.5. These signals were processed using an overlapping segmentation strategy creating a dense sequence of inputs that mimics a continuous neural stream. Specifically, a sliding window with a length of 100 ms was advanced across the signal using a step size of 5 ms, a temporal resolution aligned with the 200 Hz low-pass filter applied during neural preprocessing. Each window was labeled according to the speech state corresponding to the final time point within the window, ensuring causal alignment and no future information was used. The classifiers trained on the discrete segments were then benchmarked against this continuous evaluation set to assess temporal consistency and their generalizability to live decoding scenarios. No future neural information beyond the current prediction time point was used during continuous decoding. It should be noted that the 5 ms step size resulted in 95% overlap between adjacent windows (95 of 100 ms shared), introducing substantial temporal dependence among consecutive predictions. To mitigate the effect of this dependence on performance estimation, decoding accuracy was computed at the sentence level—that is, one accuracy value was obtained per sentence trial by comparing the full sequence of window-level predictions against the corresponding ground-truth labels. This sentence-level evaluation avoids treating highly correlated adjacent windows as independent samples.

### 2.7 The decoding accuracy of different brain regions

To investigate the spatial distribution of decoding information and quantify the specific contribution of distinct anatomical structures, a region-specific performance analysis was conducted. Neural signal channels were partitioned into three anatomically defined subsets: electrodes located within the “speech-related region,” those encompassing general “cortical” areas, and those situated in “subcortical” structures. For each subset, a decoding model was trained using solely the isolated signals from that specific region. These region-restricted models were then subjected to the identical evaluation protocol used for the full-feature model. By benchmarking the performance of these spatially constrained models against the baseline model, which retained a complete view of all neural signal channels, we assessed the necessity and sufficiency of each anatomical region for accurate speech state decoding.

### 2.8 Robustness and channel ablation analysis

To rigorously evaluate the stability of the proposed model against sensor failure and to determine its dependency on specific spatial features, a systematic robustness verification was conducted using two distinct channel ablation strategies. First, to simulate scenarios of random electrode malfunction or varying signal quality common in clinical settings, a stochastic channel dropout experiment was implemented. In this test, subsets of the recording channels were randomly dropped at increasing levels of severity, specifically excluding 10, 30, and 50% of the total available channels from the input features. Second, to assess the model’s generalization capability and its reliance on primary anatomical biomarkers, a critical region exclusion test was performed. In this scenario, all channels located within classical speech-related cortical regions—defined as the angular gyrus, frontal gyri (superior, middle, and inferior), temporal gyri (superior, middle, and inferior), and precentral gyrus, based on established models of the language network (Hickok and Poeppel, 2007; Price, 2012), were systematically removed. This targeted ablation served to verify whether the model gained decoding performance by leveraging neural information from primary speech network.

### 2.9 Contribution of different anatomical brain regions

From a holistic perspective, we also investigated the functional involvement of various brain regions by incorporating all recorded channels as model inputs. To quantify the diagnostic importance of each signal, we utilized a gradient-based saliency scoring mechanism derived from Class Activation Mapping (CAM) (Lucas et al., 2022). For a given SEEG input $x_{i,j}\in {\mathbb{R}}^{T\times C}$ (where T and C denote temporal length and channel count, respectively), the saliency ζ for channel C is formulated as follows:


$$\zeta (c)=\frac{1}{T}\times \sum_{i=1}^{T}|\frac{\partial ℒ}{\partial x_{i,c}}|$$

In this expression, ℒ represents the partial derivative of the model’s loss function. By computing the time-averaged partial derivative of the loss with respect to the input signal, we assessed how information fluctuations in specific channels modulate decoding accuracy. These saliency scores were subsequently normalized to a range of [0, 1], providing a standardized metric to elucidate the regional contributions within the Mandarin decoding framework.

## 3 Results

### 3.1 Speech state identification performance

**Figure 4:**
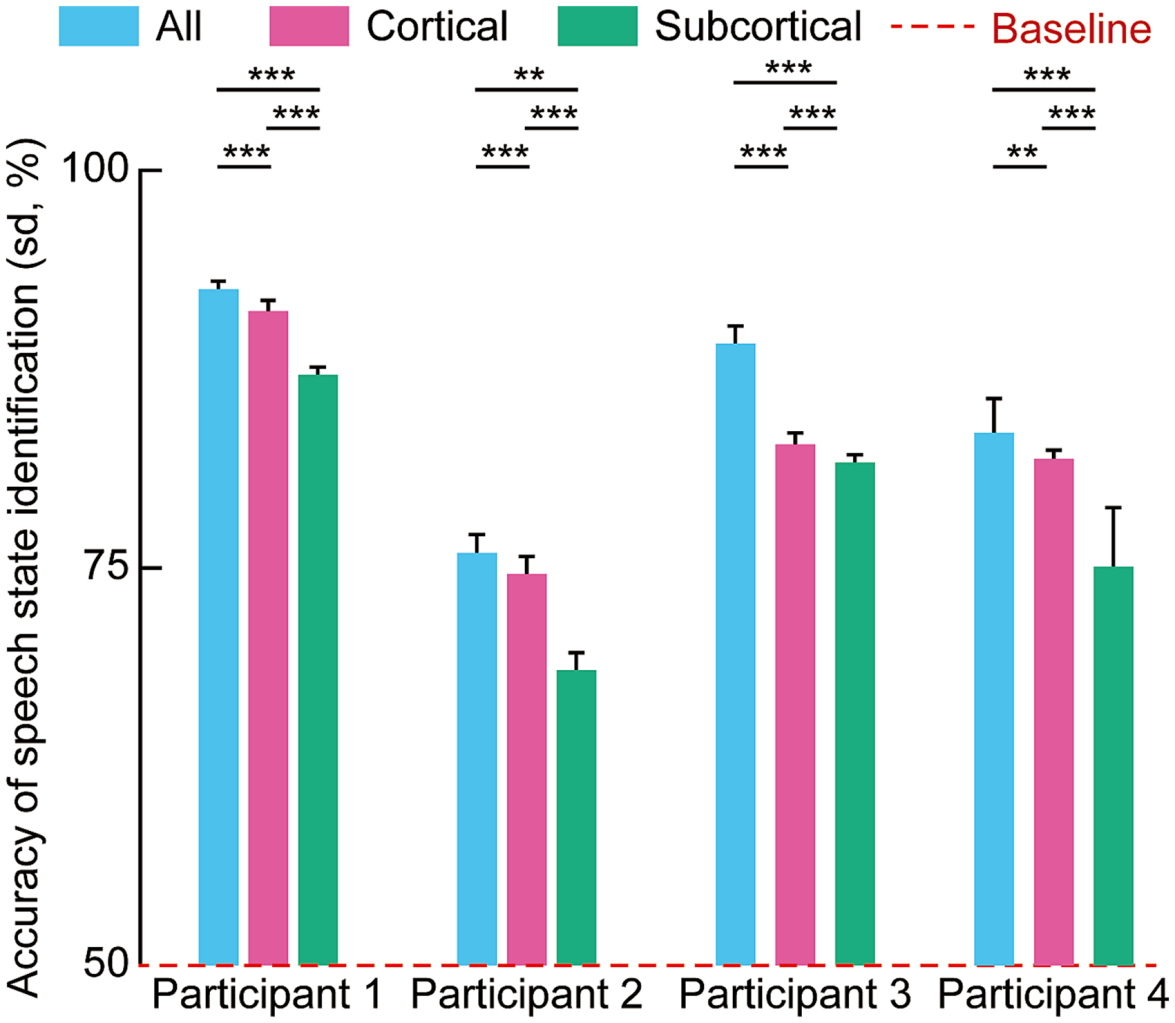
Accuracy of speech state identification across four participants using broadband (raw) signals. Bar plots show the accuracy (mean ± SD) of speech state identification for each participant under three conditions: All (combined cortical and subcortical signals), cortical-only and subcortical-only. Across participants, models incorporating both cortical and subcortical SEEG signals achieved higher decoding accuracy than models using cortical or subcortical signals alone. To evaluate the differences among the three experimental conditions, a one-way analysis of variance (ANOVA) was performed independently for each of the four participants. Upon obtaining significant omnibus results (*p* < 0.05), Tukey’s Honest Significant Difference (HSD) test was employed for *post-hoc* pairwise comparisons (*p < 0.05, **p < 0.01, ***p < 0.001).

To evaluate the contribution of different neural sources to decoding efficacy, we analyzed the accuracy of speech state identification across four participants. As illustrated in Figure 4, decoding accuracies for all participants across all spatial configurations, specifically models utilizing channels in the cortical regions, channels in subcortical structures, and the integration of both cortical and subcortical signals, exceeded the 50% chance-level baseline.

Statistical analysis using a one-way analysis of variance (ANOVA) and *post-hoc* Tukey’s Honest Significant Difference (HSD) tests revealed significant differences in performance based on the signal source. For all four participants, the combined model achieved numerically higher decoding accuracy compared to models restricted to a single anatomical domain. For instance, in Participant 1, the combined signal model reached an identification accuracy of approximately 90%. Furthermore, decoding based on cortical channels yielded significantly higher accuracy compared to subcortical channels across the entire cohort.

**Figure 5:**
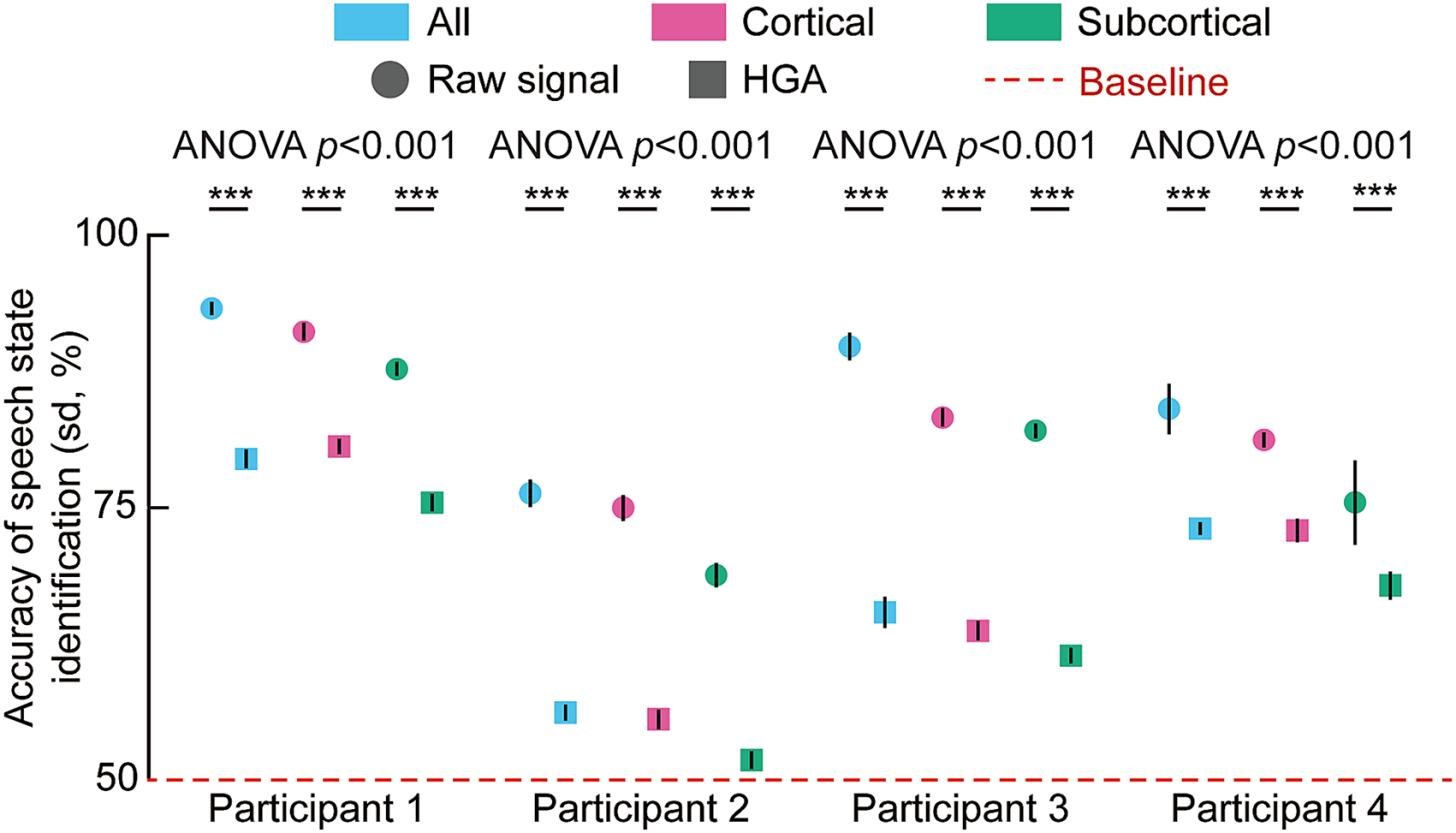
Accuracy of speech state identification across signal types. This figure illustrates the decoding performance (mean ± SD) for four participants under three regional configurations: All (blue), Cortical (pink), and Subcortical (green). Data points represent the mean identification accuracy, where circles denote models trained on raw signals and squares denote those trained on HGA, relative to a 50% chance-level baseline (red dashed line). Statistical evaluation involved a two-way ANOVA for each participant, with the ANOVA *p*-values shown at the top of each panel specifically indicating the main effect of signal type. To compare raw signal versus high gamma activity performance within each specific region, a Bonferroni-corrected simple effects analysis (*post-hoc* comparisons) was subsequently performed. Statistical significance for the ANOVA is provided above each participant, while *post-hoc* comparisons are indicated by *p < 0.05, **p < 0.01, ***p < 0.001, and n.s. for non-significant differences.

We further investigated the impact of feature extraction methods on decoding performance by comparing models trained on raw signals with those trained on HGA. This comparison was conducted across the three spatial configurations (cortical, subcortical, and their combination) for each participant (Figure 5). The results demonstrate a consistent superiority of raw signal features over HGA across all tested conditions. One-way ANOVA indicated significant differences for each participant, and *post-hoc* tests confirmed that raw signals yielded higher identification accuracy than HGA within every spatial domain. While both feature types supported decoding performance above the 50% baseline, the raw signal provided a substantial gain in accuracy. This trend was observed regardless of whether the signals originated from cortical or subcortical electrodes. We note that for Participant 1, the cortical-only HGA model yielded marginally higher accuracy than the combined HGA model, likely because subcortical signals exhibited lower signal-to-noise ratios under HGA filtering, and their inclusion introduced noise that offset the spatial information gain. This exception was not observed under broadband conditions, where subcortical low-frequency components provided complementary information.

Additionally, the performance hierarchy observed in the spatial analysis remained stable across feature types: the simultaneous integration of cortical and subcortical channels provided the most robust identification, followed by cortical-only and then subcortical-only models. These findings suggest that subcortical signals contain decodable information relevant to speech state identification, the raw signal provides a more informative feature set for decoding than HGA alone.

### 3.2 Continuous decoding ability

**Figure 6:**
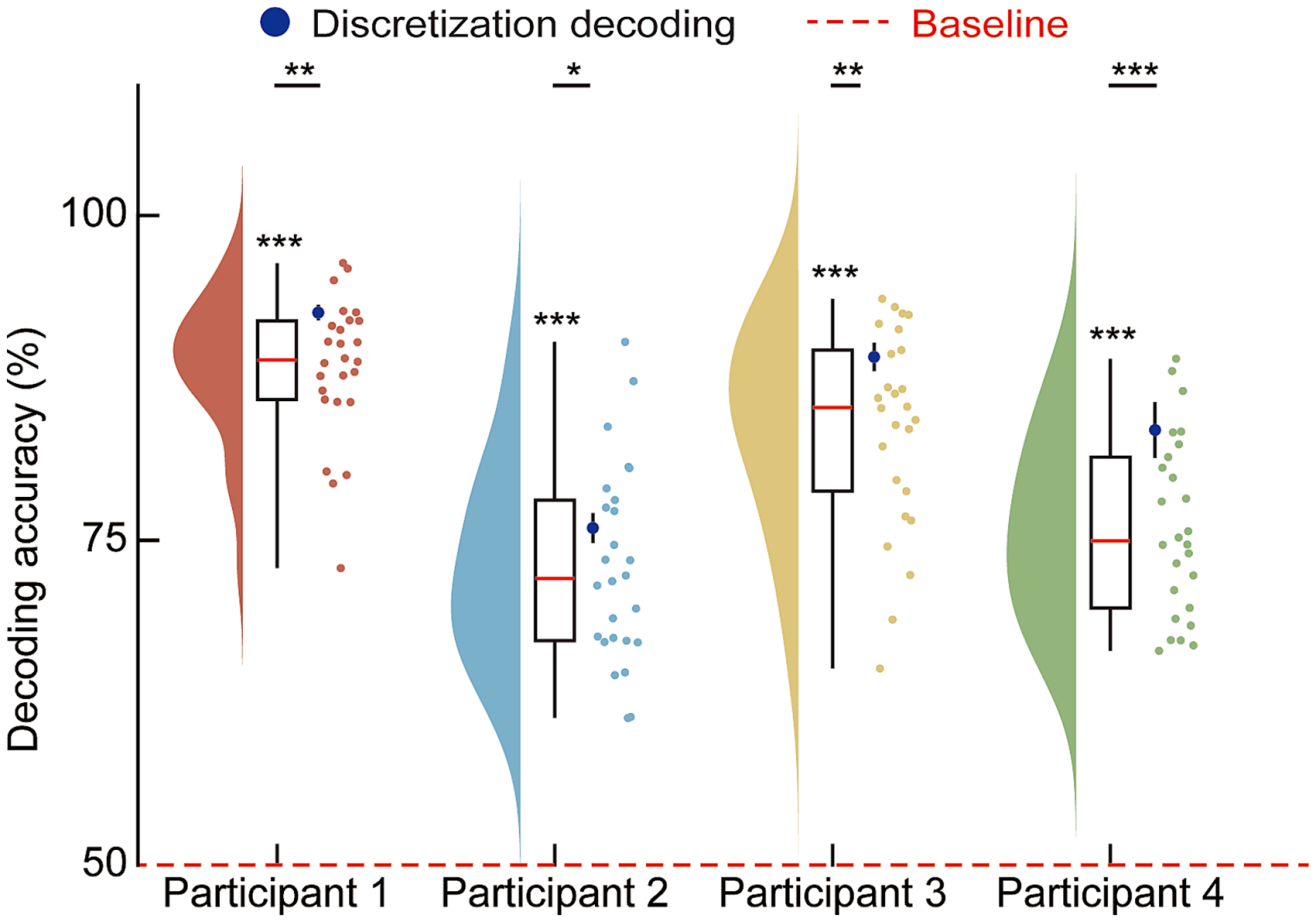
Ability of continuous decoding performance across participants using broadband (raw) signals. Continuous decoding performance for four participants compared against discretization decoding and chance levels. Each participant’s continuous decoding performance is visualized using a raincloud plot, where the shaded area on the left represents the kernel density estimation of decoding accuracy, the central box plot indicates the median and interquartile range (IQR), and the individual scatter points on the right depict the raw accuracy values of the test samples. All participants achieved continuous decoding accuracies significantly above the 50% chance-level baseline (one-sample *t*-test). The blue circular markers and associated error bars represent the mean and standard deviation (SD) of the discretization decoding results. Comparative analysis shows that while continuous decoding performance remained high, it was significantly lower than the offline discretization performance for all participants. Asterisks denote statistical significance: *p < 0.05, **p < 0.01, ***p < 0.001.

To evaluate the practical feasibility of the decoding model in a real-time context, we assessed continuous decoding performance for all four participants and compared these results against the previously established discretization decoding performance and a 50% theoretical chance-level baseline. Statistical analysis revealed that all participants achieved continuous decoding accuracies significantly above the 50% chance-level baseline (one-sample *t*-test, *p* < 0.001 for all participants). As shown in Figure 6, the median accuracy for Participant 1 was the highest among the cohort, with the distribution of individual test samples predominantly clustered between 80 and 95%. Participants 3 and 4 also demonstrated high performance, with median values exceeding 75%, while Participant 2 exhibited the lowest median accuracy, with raw values distributed more broadly between 60 and 85%. A comparative analysis between continuous and discretization decoding (blue dot with error bar for four participants in Figure 6) was performed to assess performance stability. While continuous decoding performance remained high, it was found to be significantly lower than the discretization decoding performance for all four participants (independent-samples *t*-tests with Bonferroni correction for all four participants, *p* < 0.01 for participant 1, 2 and 3, *p* < 0.001 for participant 4).

In summary, these results demonstrate that the model was able to identify speech states in a continuous data stream with accuracy above chance level. Although the transition from discretized to continuous decoding results in a measurable decrease in accuracy, the performance levels remain consistently and significantly above chance across all participants, suggesting that the decoding framework may be adaptable to real-time implementations, pending further validation.

### 3.3 Robustness of decoding model with sparsity signal

**Figure 7:**
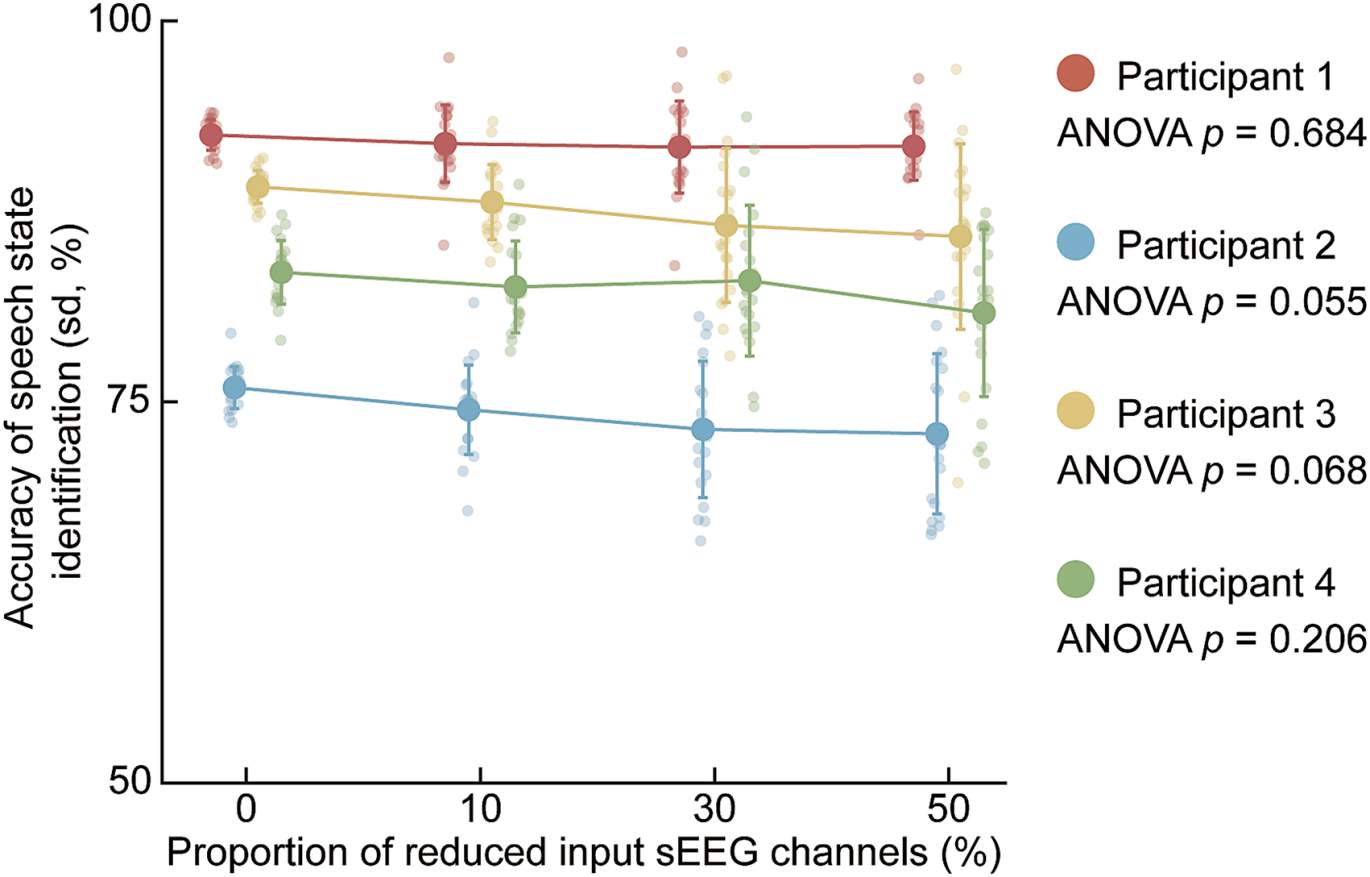
Impact of randomly reducing the proportion of input SEEG channels on speech state identification accuracy. The figure illustrates the decoding accuracy (mean ± SD) for four participants (Participants 1–4) across varying proportions of randomly removed channels (0, 10, 30, and 50%). Large dots represent the mean accuracy, and smaller dots represent individual random seed (*n* = 20). A one-way ANOVA was performed for each participant to assess the statistical significance of the differences in accuracy across the varying channel reduction levels. For all participants, the resulting *p*-values were non-significant, suggesting that the decoding performance remains robust even when the number of input electrodes is reduced by up to 50%.

**Figure 8:**
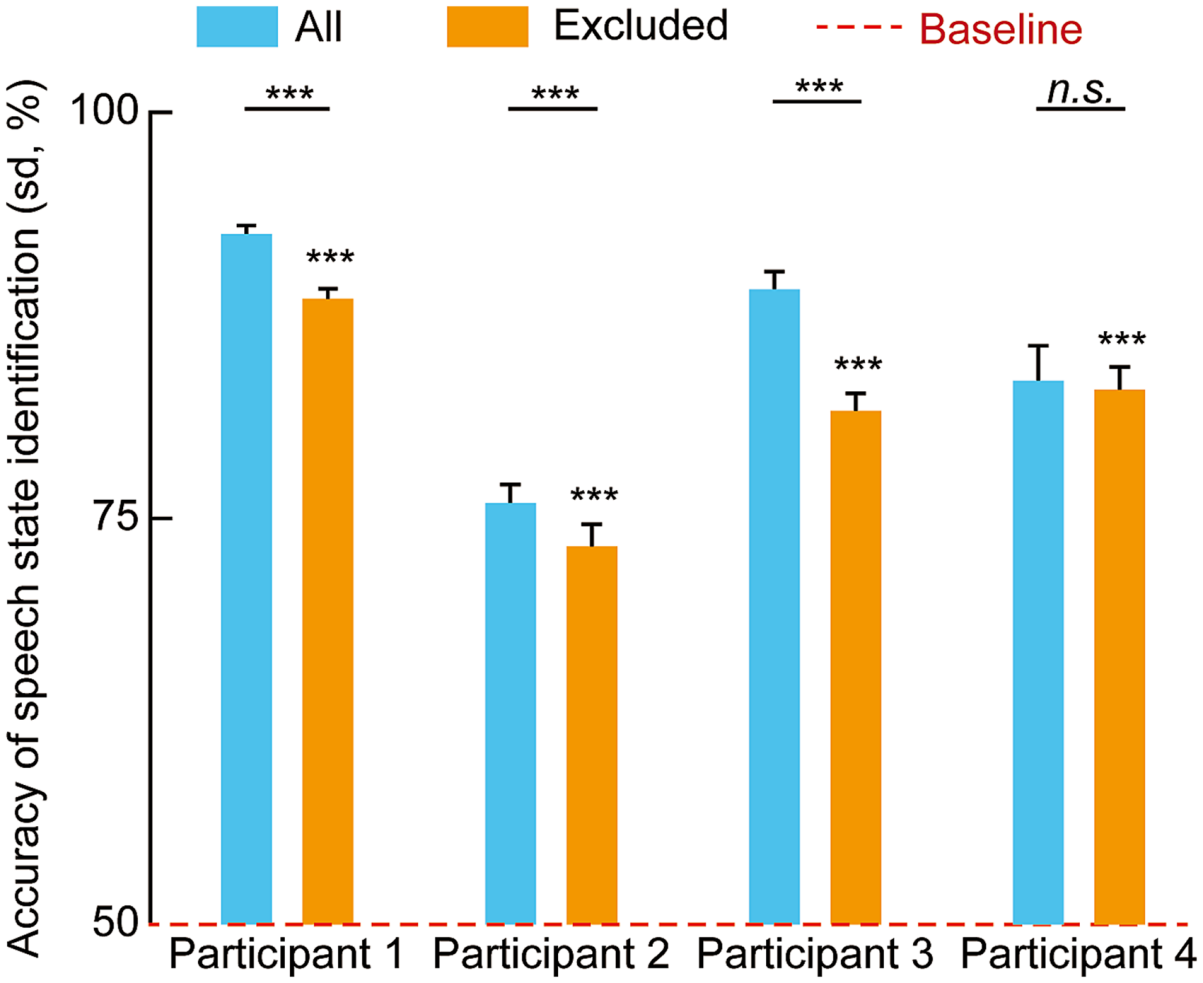
Impact of excluding classical speech-related regions on speech state identification accuracy. This figure compares the decoding performance across four participants under two conditions: “All” (blue bars), utilizing neural signals from all recorded channels, and “Excluded” (orange bars), where channels from regions traditionally associated with speech processing—including the angular gyrus, frontal gyrus, temporal gyrus, and precentral gyrus—were removed. The red dashed line represents the theoretical chance level (50%), and error bars indicate the standard deviation (SD). Statistical significance is denoted by asterisks above individual bars for comparisons against the baseline and by horizontal brackets for pairwise comparisons between conditions (independent samples *t*-test). Asterisks denote statistical significance: *p < 0.05, **p < 0.01, ***p < 0.001.

To assess the robustness of the decoding framework against signal sparsity and electrode loss, we conducted two distinct evaluations: the random reduction of input channels (Figure 7) and the targeted exclusion of classical speech-related brain regions (Figure 8).

As illustrated in Figure 7, the impact of randomly reducing the proportion of input SEEG channels on speech state identification accuracy was evaluated for all four participants. The proportion of removed channels was varied across four levels: 0, 10, 30, and 50%. For all participants, the mean decoding accuracy exhibited a stable trajectory across these levels, with mean performances remaining well above the 50% chance-level baseline even at 50% channel reduction. A one-way ANOVA performed for each participant confirmed that the differences in accuracy across the varying levels of channel removal were not statistically significant (Participant 1: *p* = 0.684; Participant 2: *p* = 0.055; Participant 3: *p* = 0.068; Participant 4: *p* = 0.206). These results suggest that decoding performance remained relatively stable under channel reduction of up to 50% within the present dataset.

We further evaluated the model’s resilience by comparing the “All” condition (utilizing all recorded channels) against an “Excluded” condition, where channels from regions traditionally associated with speech processing, including the Angular Gyrus, Frontal Gyrus, Temporal Gyrus, and Precentral Gyrus, were removed (Figure 8). In the “Excluded” condition, decoding accuracies for all four participants remained significantly higher than the 50% theoretical chance level (*p* < 0.001).

Independent samples *t*-tests were employed to compare the two conditions for each participant. For Participants 1, 2, and 3, the exclusion of classical speech regions led to a statistically significant decrease in decoding accuracy (*p* < 0.001). However, for Participant 4, the difference between the “All” and “Excluded” conditions did not reach statistical significance (n.s.). Despite the performance declines observed in most participants, the identification accuracies in the absence of classical speech areas remained high, exceeding 70% in all participants.

### 3.4 Contributions of different brain regions

**Figure 9:**
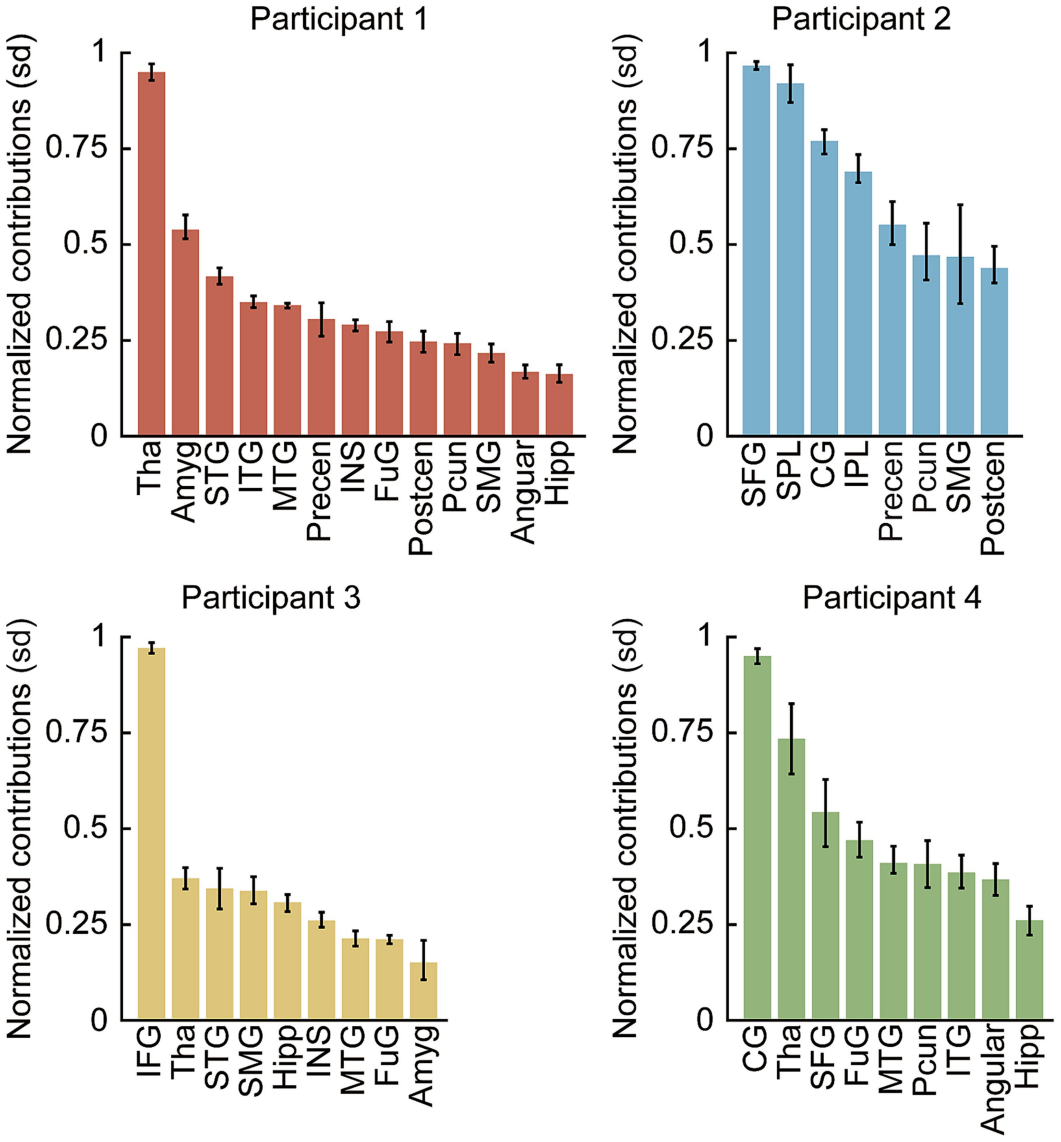
Contributions of different brain regions to speech state identification. This figure illustrates the normalized contributions (mean ± SD) of various brain regions for each of the four participants. The regions are ranked in descending order based on their mean contribution to the decoding model. For Participant 1, the Thalamus (Tha) shows the highest contribution, whereas the Superior Frontal Gyrus (SFG) and Cingulate Gyrus (CG) are the primary contributors for Participant 2 and Participant 4, respectively. In Participant 3, the Inferior Frontal Gyrus (IFG) stands out as the most significant region. Tha, Thalamus; Amyg, Amygdala; STG, Superior Temporal Gyrus; ITG, Inferior Temporal Gyrus; MTG, Middle Temporal Gyrus; Precen, precentral gyrus; Postcen, Postcentral Gyrus; INS, Insula; FuG, Fusiform Gyrus; Pcun, Precuneus; SMG, Supramarginal Gyrus; Angular, Angular Gyrus; Hipp, Hippocampus; SFG, Superior Frontal Gyrus; IFG, Inferior Frontal Gyrus; SPL, Superior Parietal Lobule; IPL, Inferior Parietal Lobule; CG, Cingulate Gyrus.

To evaluate the relative importance of distinct anatomical areas in the speech decoding models, the normalized contributions of various brain regions were analyzed for each participant (Figure 9). The evaluated regions were ranked in descending order based on their mean contribution weight. The analysis revealed distinct, participant-specific profiles of regional involvement. The primary contributing region varied across the cohort: the thalamus exhibited the highest contribution for Participant 1, the superior frontal gyrus for Participant 2, the inferior frontal gyrus for Participant 3, and the cingulate gyrus for Participant 4.

Despite this inter-participant variability in the single most informative region, notable consistencies were observed across the cohort. The thalamus exhibited relatively high model sensitivity in three of the four participants (Participants 1, 3, and 4). Concurrently, specific cortical areas, particularly within the frontal and limbic regions (SFG, IFG, and CG), consistently demonstrated dominant contributions in individual models. Conversely, regions such as the hippocampus and angular gyrus consistently exhibited the lowest normalized contributions across multiple participants. Notably, Participant 2 exhibited a distinct profile in which subcortical regions contributed minimally to the decoding model, with the highest contributions instead concentrated in superior frontal and cingulate cortical areas. This participant had the fewest implanted electrodes (8 electrodes), resulting in comparatively limited subcortical sampling. The absence of prominent subcortical contributions in this case may therefore reflect insufficient spatial coverage of deep structures rather than a true lack of subcortical involvement in speech state processing.

In summary, the contribution weights indicate that speech state identification relies on a distributed network. While the dominant individual region is participant-dependent, both specific cortical areas and the subcortical thalamus consistently showed higher contribution weights within the decoding models.

## 4 Discussion

In this study, we investigated speech state identification (rest vs. overt speech) using stereotactic electroencephalography (SEEG), a sparse neural recording electrode, recordings from four participants performing a Mandarin reading task. Three key findings emerged: (1) Models incorporating both cortical and subcortical signals consistently outperformed models based on either domain alone;(2) broadband raw signals yielded superior decoding accuracy compared to high-gamma activity (HGA) features alone; and (3) the decoding framework demonstrated remarkable robustness to channel dropout and exclusion of classical language regions. These findings are consistent with the hypothesis that speech state representations involve distributed cortico-subcortical activity and can be decoded from sparse clinical SEEG recordings in individual subjects.

Given the small sample size and the variability in electrode placement, the present findings should be interpreted at the individual-participant level rather than as population-level generalizations. Specifically, because SEEG electrode placement is determined entirely by clinical needs for epilepsy monitoring, no two participants shared identical coverage of subcortical structures. The subcortical regions sampled—including the thalamus, amygdala, and hippocampus—differed in both number and spatial extent across the four individuals. Therefore, our conclusion that speech state information is distributed across a cortico-subcortical network is supported at the individual-participant level: each participant’s model independently benefited from the inclusion of whatever subcortical signals were available. However, this does not constitute evidence for a fixed, population-level network topology, which would require validation in larger cohorts with more systematic and standardized coverage.

The superior performance of combined cortical–subcortical models aligns with contemporary distributed network theories of speech production. Classical neuroanatomical models emphasized focal cortical loci, such as Broca’s area, as core speech centers (Broca, 1861). However, modern frameworks, including the dual-stream model (Hickok and Poeppel, 2007; Hickok, 2022), propose that speech production and perception rely on distributed dorsal and ventral processing streams involving temporal, frontal, parietal, and sensorimotor cortices. Increasingly, subcortical structures are recognized as integral components of this network (Hickok, 2022; Silveri, 2021; Kotz et al., 2009; Tourville and Guenther, 2011). The ability of SEEG to simultaneously sample cortical and deep structures provides a unique opportunity to capture such cross-level dynamics. Importantly, our findings suggest that speech state detection benefits from this cortico-subcortical integration. Unlike high-resolution electrocorticography (ECoG), which primarily samples superficial cortex, SEEG enables access to deep nodes within the speech network (Bartolomei et al., 2017). The present results suggest that subcortical signals may contribute additional information beyond cortical recordings alone.

The consistent emergence of the thalamus as a high-contribution region across multiple participants invites consideration of its specific functional role in speech state transitions. Several non-mutually exclusive mechanisms may account for this observation. First, the ventrolateral thalamic nuclei serve as a critical relay within the basal ganglia–thalamus-cortical motor loop, participating in speech motor initiation, sequencing, and timing control (Crosson, 2013; Parent and Hazrati, 1995). Elevated thalamic saliency in our models may therefore reflect preparatory motor signals that precede and accompany vocalization onset. Second, the thalamus—particularly the pulvinar and medial geniculate body—is implicated in sensory gating mechanisms that modulate auditory processing in anticipation of self-generated speech (Sherman, 2012; Houde and Chang, 2015). This predictive sensory suppression could generate a distinctive neural signature distinguishing speech from rest states. Third, the thalamus plays a well-established role in regulating cortical excitability and mediating transitions between attentional and arousal states (Wolff et al., 2021; Wang et al., 2025). The shift from rest to active speech production likely involves a global arousal state change that is prominently reflected in thalamic activity. Given that our SEEG recordings lack sub-nuclear spatial resolution within the thalamus, the present data cannot disambiguate among these hypotheses. Future studies combining higher-resolution thalamic recordings with task designs that independently manipulate motor, sensory, and attentional demands during speech will be necessary to dissect the thalamus’s specific contributions to speech state transitions.

The present study excluded electrode contacts localized within white matter, following the conventional assumption that white matter signals primarily reflect volume-conducted activity rather than local neuronal processing. However, recent evidence suggests that white matter recordings from intracranial electrodes may carry functionally relevant information. Given that white matter tracts serve as the structural conduits connecting cortical and subcortical nodes of the speech network, signals recorded from these pathways could potentially capture propagating neural activity that reflects inter-regional communication dynamics. Incorporating white matter channels into the decoding framework may therefore provide complementary information that further enhances speech state detection accuracy. Future work should systematically evaluate the contribution of white matter signals, both independently and in combination with gray matter recordings, to determine whether they offer incremental predictive value for speech BCI applications.

High-gamma activity has frequently been emphasized as a robust correlate of local neuronal firing and has been widely used in speech decoding studies (Leonard et al., 2024; Silva et al., 2024a; Moses et al., 2021; Anumanchipalli et al., 2019). Nevertheless, in our study, broadband raw signals consistently outperformed HGA-only features. Several factors may explain this finding. First, speech state transitions likely involve multi-frequency dynamics, including low-frequency oscillations associated with motor preparation, attention, and large-scale coordination, in addition to local high-frequency activity (Siegel et al., 2012; Fries, 2015; Crone et al., 1998; Buzsaki and Wang, 2012; Houde and Chang, 2015). Broadband representations allow the model to exploit cross-frequency interactions and phase–amplitude coupling that may carry additional discriminative information (Lu et al., 2022). Second, subcortical local field potentials often exhibit prominent low-frequency components that would be attenuated when restricting analysis to HGA. Third, recent work has cautioned that speech-related mechanical or acoustic artifacts may contaminate high-frequency bands in intracranial recordings (Bush et al., 2022). These observations suggest that speech state detection, particularly in heterogeneous SEEG configurations, may benefit from preserving multi-scale neural information rather than constraining feature extraction to predefined frequency bands. End-to-end architectures operating on minimally processed broadband signals may therefore be advantageous in clinical BCI contexts.

Regarding model architecture, recent Transformer-based pre-trained models such as BrainBERT (Wang et al., 2023) have shown strong representation learning capabilities for intracranial EEG. However, several factors motivated the use of a lightweight CNN in the present study. First, pre-trained neural models were primarily developed on high-density ECoG data with standardized electrode configurations, whereas SEEG electrode placement varies entirely across participants, posing fundamental challenges for cross-participant transfer. Second, the binary classification task (speech vs. rest) has low computational complexity, for which a CNN provides sufficient capacity without the overhead of large models. Third, real-time BCI deployment demands low-latency inference on resource-constrained hardware. Fourth, the limited per-participant data (300 trials) are insufficient for fine-tuning large pre-trained models without overfitting. As standardized SEEG datasets grow and cross-participant pre-training strategies mature, integrating foundation models with sparse intracranial recordings represents a promising future direction.

The present study was conducted exclusively with Mandarin-speaking participants, and cross-linguistic generalizability remains to be established. However, speech state detection—distinguishing active speech production from rest—depends primarily on general motor and physiological neural signatures (motor planning, respiratory coordination, thalamic activation) rather than on language-specific phonological or semantic features (Hickok and Poeppel, 2007). This is supported by evidence that cortical articulatory representations are shared across languages in bilingual speakers (Silva et al., 2024a; Silva et al., 2024b). Nonetheless, language-specific factors such as tonal contours in Mandarin could influence the spectral dynamics of speech-related neural activity, and empirical cross-linguistic validation using publicly available SEEG datasets remains an important direction for future work. Beyond its technical significance, autonomous speech state detection carries ethical implications for the design of future speech BCIs. A key concern in neural speech decoding is the risk of unintended or involuntary decoding of private cognitive processes (Yuste et al., 2017). A reliable speech state detector functions as a gating mechanism that restricts neural decoding to periods of active, intended speech production, thereby preserving the user’s communicative autonomy and aligning with ethical frameworks for neural interfaces.

Several limitations merit consideration. First, the sample size was modest (*N* = 4), and electrode placement was entirely clinically determined, resulting in substantial inter-participant variability in both cortical and subcortical coverage. Although within-participant performance was robust, the distributed network conclusion is drawn from individual-level analyses and cannot be directly generalized to a canonical population-level architecture. Broader generalizability requires validation in larger, multi-center cohorts with more standardized electrode coverage. Second, the study focused on overt speech. Although overt, mouthed, and imagined speech share partially overlapping neural substrates, their signal characteristics differ (Soroush et al., 2023). Finally, although streaming simulations demonstrated promising performance, full real-time closed-loop implementation will require further optimization in terms of latency, computational efficiency, and long-term stability. Additionally, the present study collected all data within a single experimental session, precluding evaluation of longitudinal practice effects. While recent work (Bhadra et al., 2025) reported that imagined speech decoding accuracy improved with repeated multi-session training, such effects are less expected for overt speech state detection, which relies on involuntary motor and physiological signatures of an already automatized skill. Nevertheless, multi-session longitudinal studies will be important to assess the long-term stability of speech state decoding and to characterize potential adaptation effects in extended BCI use. Future work should explore speech state detection across different speech modalities, investigate transfer learning approaches to address inter-participant variability, and develop adaptive algorithms for long-term BCI stability. The integration of speech state detection with content decoding modules represents a particularly promising direction for clinically viable speech BCIs.

## 5 Conclusion

This study demonstrates that speech state can be reliably decoded from sparse SEEG recordings in Mandarin-speaking participants. Integrating cortical and subcortical signals significantly improves decoding performance relative to single-domain models. The decoding framework remains robust under channel dropout and exclusion of classical language regions, suggesting the reducing reliance on specific anatomical regions. These findings provide preliminary support for the feasibility of developing speech activity detection modules based on clinical SEEG recordings. Such modules may serve as foundational components of future speech brain–computer interfaces, particularly in patient populations where electrode placement cannot be optimized solely for communication purposes.

## Data Availability

The raw data supporting the conclusions of this article will be made available by the authors, without undue reservation.

## Supplementary material

SUPPLEMENTARY FIGURE S1 Objective assessment of acoustic contamination in the neural signal for four participants. The heatmap on the left represents the contamination matrix for the neural data of each participant. Brighter colors indicate higher correlation. The graph on the right represents a statistical assessment of acoustic contamination in the neural data for each patient. It compares the average value of the diagonal of the contamination matrix to the distribution of this value in 10,000 randomly shuffled contamination matrices (*p*-values are displayed within the figures, one-sided).
