# Supplementary figures and images for: Distributed cortico-subcortical networks enable robust speech state detection from sparse intracranial recordings
Source: Front Neurosci. 2026 May 8;20:1816455. doi: 10.3389/fnins.2026.1816455 (PMC13194996; doi:10.3389/fnins.2026.1816455)

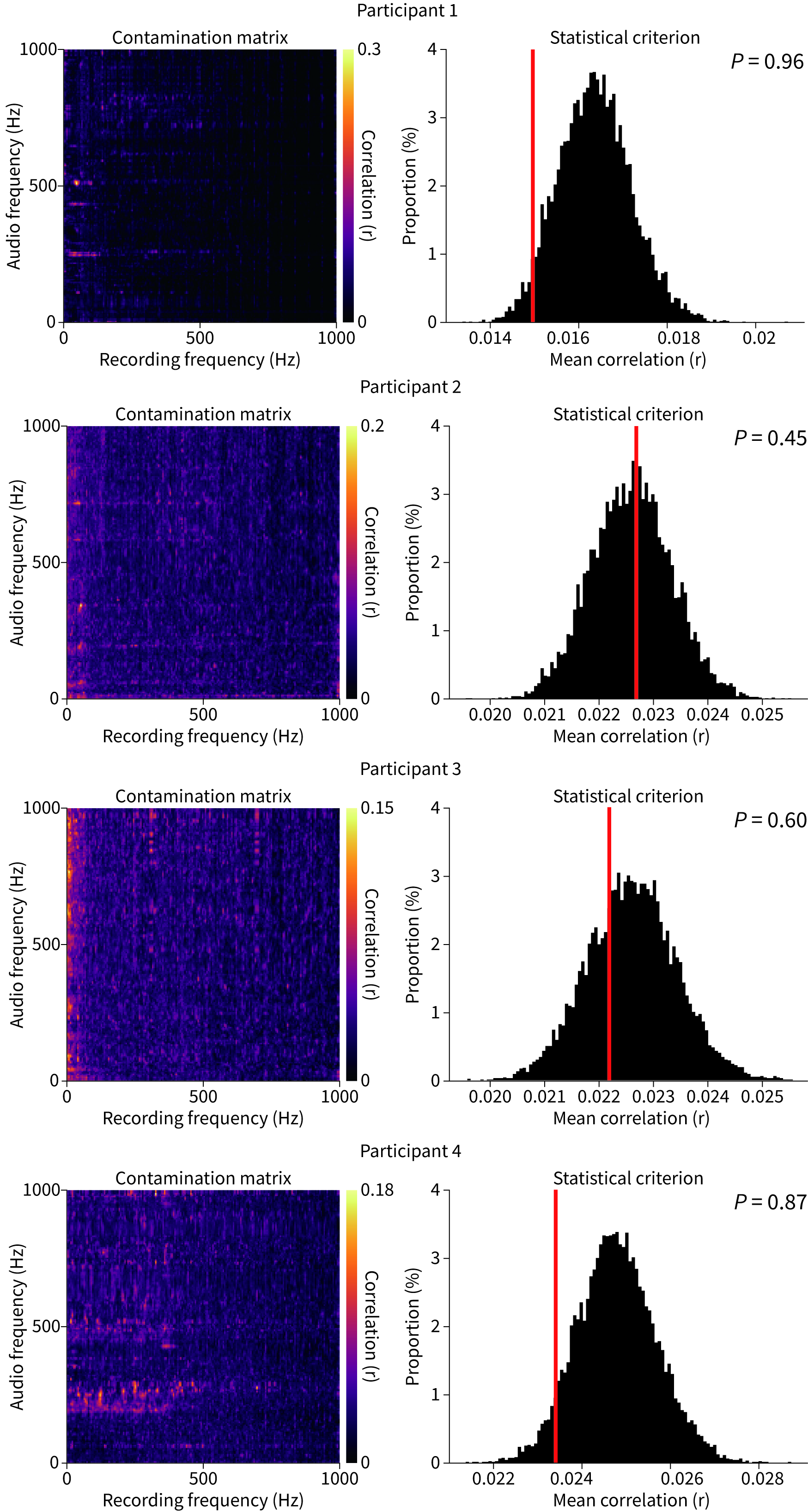

Supplement: SUPPLEMENTARY FIGURE S1 — Objective assessment of acoustic contamination in the neural signal for four participants. The heatmap on the left represents the contamination matrix for the neural data of each participant. Brighter colors indicate higher correlation. The graph on the right represents a statistical assessment of acoustic contamination in the neural data for each patient. It compares the average value of the diagonal of the contamination matrix to the distribution of this value in 10,000 randomly shuffled contamination matrices (p-values are displayed within the figures, one-sided). [file Image_1.tif]
